# Validation of the severity index by cardiac catheterization and Doppler echocardiography in patients with aortic sclerosis and stenosis

**DOI:** 10.1186/1476-7120-4-12

**Published:** 2006-03-21

**Authors:** David M Shavelle, Nediljka Buljabasic, Junichiro Takasu, Ashkan Babaie, Joseph Rosales, Matthew J Budoff, Kevin D O'Brien

**Affiliations:** 1 Los Angeles BioMedical Research Institute at Harbor-UCLA Research, Division of Cardiology, Torrance, CA, USA; 2 Division of Cardiology, University of Washington, Seattle, WA, USA

## Abstract

The severity index is a new echocardiographic measure that is thought to be an accurate indicator of aortic leaflet pathology in patients with AS. However, it has not been validated against cardiac catheterization or Doppler echocardiographic measures of AS severity nor has it been applied to patients with aortic sclerosis. The purposes of this study were to compare the severity index to invasive hemodynamics and Doppler echocardiography across the spectrum of calcific aortic valve disease, including aortic sclerosis and AS. 48 patients with aortic sclerosis and AS undergoing echocardiography and cardiac catheterization comprised the study population. The aortic valve leaflets were assessed for mobility (scale 1 to 6) and calcification (scale 1 to 4) and the severity index was calculated as the sum of the mobility and calcification scores according to the methods of Bahler et al. The severity index increased with increasing severity of aortic valve disease; the severity indices for patients with aortic sclerosis, mild to moderate AS and severe AS were 3.38 ± 1.06, 6.45 ± 2.16 and 8.38 ± 1.41, respectively. The aortic jet velocity by echocardiography and the square root of the maximum aortic valve gradient by cardiac catheterization correlated well with the severity index (r = 0.84, p < 0.0001; r = 0.84, p < 0.0001, respectively). These results confirm that the severity index correlates with hemodynamic severity of aortic valve disease and may prove to be a useful measure in patients with aortic sclerosis and AS.

## Background

Aortic stenosis (AS) and aortic sclerosis are common findings in elderly patients, affecting 50% of those over the age of 84 years [1]. Echocardiography provides an accurate noninvasive assessment of disease severity and progression [2,3]. Studies have found that the presence of aortic valve calcium is associated with increased disease severity and predicts more rapid disease progression [4]. Bahler et. al. proposed that the severity index, a measure of both aortic valve leaflet calcium and mobility, was an accurate indicator of aortic leaflet pathology in patients with AS [5]. However, this new echocardiographic score has not been validated against cardiac catheterization measures of AS severity. Moreover, this index has not been validated against either cardiac catheterization or Doppler echocardiography in patients with aortic sclerosis. Therefore, the purposes of this study were to compare the severity index to invasive hemodynamics and Doppler echocardiography across the spectrum of calcific aortic valve disease, including aortic sclerosis and AS.

## Methods

### A. Study population

48 patients with aortic valve disease (aortic sclerosis and AS) identified by echocardiography with subsequent referral for invasive hemodynamic assessment of AS severity comprised the study population. All patients were referred for echocardiography by their treating physicians after a systolic murmur was detected. Patients underwent standard left and right heart catheterization for assessment of AS severity within one month of the echocardiogram. The institutional review board of Harbor-UCLA Research and Education Institute approved the study protocol. At the time of cardiac catheterization, information was obtained regarding the presence of traditional cardiovascular risk factors, including hypertension, family history of premature coronary artery disease, hyperlipidemia, smoking, and diabetes mellitus.

Patients were classified as having hypertension if they were receiving anti-hypertensive medications or had known but untreated hypertension (systolic blood pressure >140 mm Hg or diastolic blood pressure > 90 mm Hg). Family history of coronary artery disease was defined as premature coronary artery disease (occurring in men age less than 45 years and women age less than 55 years) in a first-degree relative. Hyperlipidemia was defined as use of cholesterol lowering medication or, in the absence of cholesterol lowering medication use, as having a total serum cholesterol >240 mg/dL. Smoking was defined as the use of >10 cigarettes/day. Patients receiving insulin or oral hypoglycemic agents were classified as having diabetes mellitus.

### B. Cardiac catheterization

Standard retrograde left and right heart catheterization was performed to evaluate AS severity. The left ventricle was entered by the retrograde approach. Left ventricular and aortic pressures were measured simultaneously using a 6 French pigtail catheter within the left ventricle, and a second 6 French pigtail catheter positioned in the ascending aorta. Computer assisted measurements of the peak-to-peak, maximum and mean gradients were obtained. Cardiac output was measured by the Fick method. Aortic valve area was calculated using the Gorlin equation [6].

Aortic sclerosis was defined as the presence of aortic valve leaflet calcification or thickening (increased echogenicity) and no restriction to leaflet excursion by echocardiography and a maximum gradient by catheterization of less than 15 mm Hg. Mild AS was defined as a maximum gradient by catheterization of greater than 15 mm Hg and less than 36 mm Hg. Moderate AS was defined as a maximum gradient by catheterization of greater than 36 mm Hg and less than 64 mm Hg. Severe AS was defined as a maximum gradient by catheterization of greater than 64 mm Hg. For the statistical analysis, patients with mild AS and moderate AS were included as one group. Patients could be grouped in similar categories using the aortic valve area by the Gorlin equation (data not shown); however, the aortic valve area was not calculated for patients with a maximum gradient by catheterization of less than 15 mm Hg.

### C. Echocardiography

Doppler-echocardiography examinations were performed on a Hewlett-Packard 77020 AC echocardiographic scanner (Hewlett-Packard, Palo Alto, CA). The peak instantaneous transvalvular aortic jet velocity was determined by interrogating the aortic valve with continuous wave Doppler from multiple acoustic windows in order to obtain the highest jet velocity. Mean Doppler velocities were calculated by averaging the instantaneous Doppler gradients throughout the ejection period using an on-line quantification package. Three cardiac beats were averaged and the spectral display velocity curve was traced by hand. Anatomic measurements of the diameter of the left ventricular outflow tract were made from the two-dimensional parasternal long-axis view, parallel and adjacent to the aortic valve plane.

AS was defined as the presence of a peak instantaneous transaortic jet velocity of ≥ 2.0 m/sec and restriction to valve leaflet opening in the presence of normal left ventricular systolic function. Patients with AS were further classified as mild AS (transaortic jet velocity 2.0 to 3.0 m/sec), moderate AS (transaortic jet velocity 3.0 to 4.0 m/sec) and severe AS (transaortic jet velocity >4.0 m/sec) [2]. Aortic sclerosis was defined as the presence of aortic valve leaflet calcium or leaflet thickening and a peak instantaneous transaortic jet velocity of < 2.0 m/sec.

The aortic valve leaflets were assessed for mobility and calcification according to the methods described by Bahler et. al. [5]. The aortic valve leaflets were assessed in both the parasternal long- and short-axis views. Aortic leaflet calcification was graded according to the scale: 1 = none, 2 = ≥1 localized area of increased reflectivity but no areas of dense calcification, 3 = markedly increased reflectivity (calcification) in one leaflet but equal to or less than grade 2 changes in other leaflets, 4 = markedly increased reflectivity in 2 leaflets but equal to or less than grade 2 changes in the third leaflet, 5 = moderately increased reflectivity in all leaflets and 6 = severely increase reflectivity in all leaflets. Leaflet mobility was graded according to the scale: 1 = normal leaflet mobility, 2 = restriction of only 1 leaflet with normal mobility of the other leaflets or mild restriction of all leaflets, 3 = marked restriction of 2 leaflets or moderate restriction of all leaflets, and 4 = almost no mobility of any leaflet. The calcification and mobility scores were summed to yield the severity index.

### C. Statistical analyses

Statistical analyses were performed using SAS v.6.12 (SAS Institute, Cary, NC) or GraphPad Prism, Version 3.02 (GraphPad Software, Inc., La Jolla, CA). Comparisons amongst the three groups (aortic sclerosis, mild to moderate AS and severe AS determined by cardiac catheterization) were done using one-way analysis of variance for continuous variables (age, maximum aortic valve gradient, transaortic jet velocity, severity index) and likelihood ratio chi-square for discrete variables (gender, hypertension, diabetes, hyperlipidemia, smoking, family history, aortic valve calcium, aortic valve mobility). These were overall tests to determine if any two groups differed from one another. Because cardiac catheterization gradients were not normally distributed, regression analysis with this variable was performed using square root-transformed values.

## Results

### A. Patient characteristics

For the 48 patients, the mean (± SD) age was 56.1 ± 9.6 years and 32 (67%) were male. The prevalence of cardiovascular risk factors was: hypertension 32 (67%), smoking 19 (40%), diabetes mellitus 17 (35%), hyperlipidemia 20 (42%), and family history of coronary artery disease 16 (33%). Twenty-eight patients had aortic sclerosis, 12 had mild to moderate AS and 8 had severe AS.

### B. Cardiac catheterization

**Figure 1:**
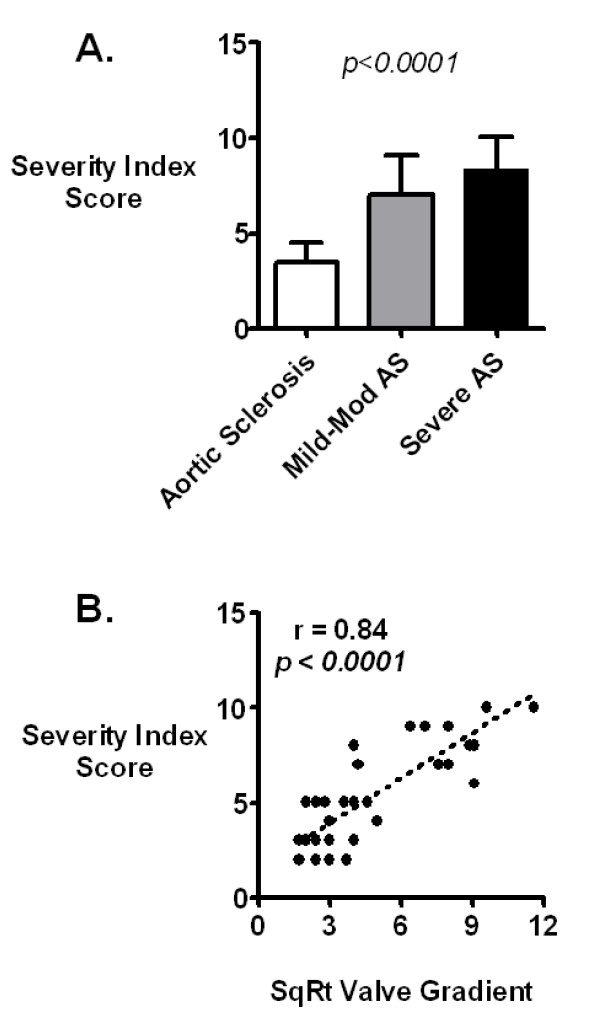
**Catheterization Gradient vs. Severity Index**. **A**. Severity index as determined by cardiac catheterization for patients with aortic sclerosis (maximum gradient <16 mmHg), mild to moderate AS (maximum gradient 16–64 mmHg) and severe AS (maximum gradient >64 mmHg). The severity index increased with increasing severity of aortic valve disease and was significantly different between the three groups (p < 0.0001 by one-way analysis of variance [ANOVA]). **B**. Correlation of Catheterization Gradient with Severity Index. There was a strong correlation between square root-transformed maximum catheterization gradient and severity index (r = 0.84).

For the entire group of patients, the maximum aortic valve gradient was 26 ± 30 mm Hg. For patients with aortic sclerosis, mild to moderate AS and severe AS, the mean (± SD) maximum aortic valve gradients were 7.0 ± 2.8, 30 ± 16 and 83 ± 24 mm Hg, respectively (Figure 1A). The square root of the maximum aortic valve gradient by catheterization correlated well with the severity index (r = 0.84, p < 0.0001, Figure 1B).

### C. Echocardiography

**Figure 2:**
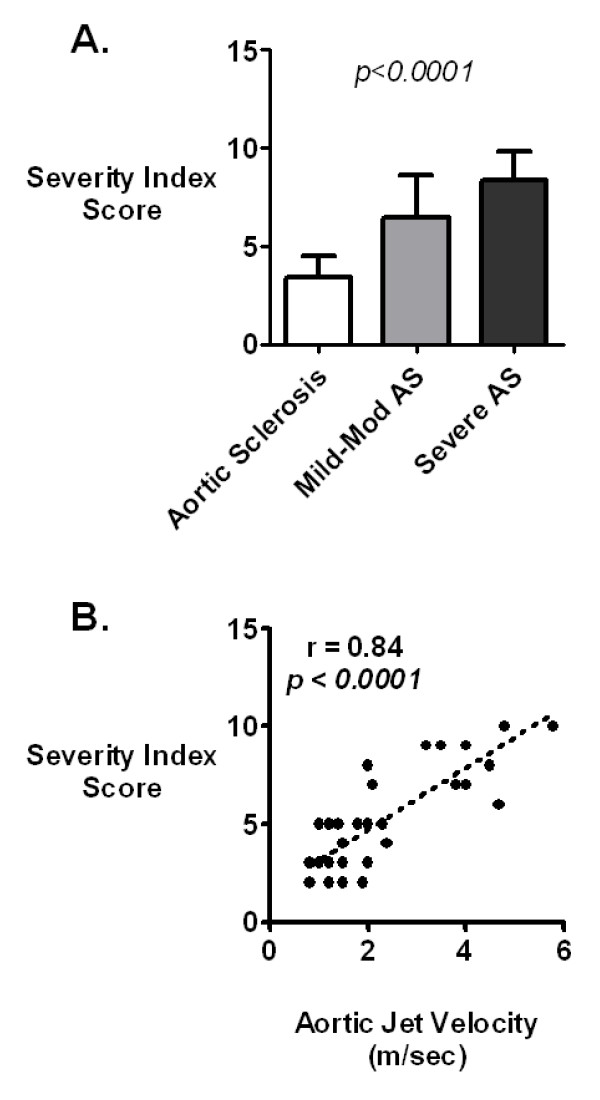
**Doppler Echocardiography vs. Severity Index**. **A**. Severity index as determined by Doppler echocardiography for patients with aortic sclerosis (aortic jet velocity <2.0 m/sec), mild to moderate AS (aortic jet velocity 2.0–4.0 m/sec) and severe AS (aortic jet velocity >4.0 m/sec). The severity index increased with increasing severity of aortic valve disease and was significantly different between the three groups (p < 0.0001 by one-way [ANOVA]). **B**. Correlation of maximum Doppler aortic jet velocity with Severity Index. There was a strong correlation between aortic jet velocity and severity index (r = 0.84).

**Figure 3:**
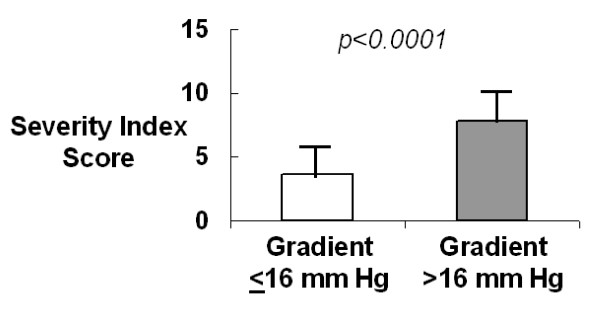
**Severity Index in Patients with a Low (<16 mm Hg) and High (≥16 mm Hg) Maximum Aortic Gradient**. The severity index was significantly higher in patients with a high maximum aortic valve gradient (>16 mm Hg) compared to patients with a low maximum aortic valve gradient (≤16 mm Hg), 7.80 ± 1.78 and 3.63 ± 1.35, respectively (p < 0.0001 by one-way [ANOVA]).

For the entire group of patients, the mean (± SD) transaortic jet velocity by echocardiography was 1.5 ± 0.4 m/sec. For patients with aortic sclerosis, mild to moderate AS and severe AS, the mean (± SD) transaortic jet velocities was 1.29 ± 0.27, 2.65 ± 0.70 and 4.54 ± 0.60 m/sec, respectively. The severity index for the entire group of patients was 5.0 ± 2.5. The severity index increased with increasing severity of aortic valve disease and was significantly different among each patient group; the severity indices for patients with aortic sclerosis, mild to moderate AS and severe AS were 3.38 ± 1.06, 6.45 ± 2.16 and 8.38 ± 1.41, respectively (Figure 2A). Aortic jet velocity correlated well with the severity index (r = 0.84, p < 0.0001, Figure 2B). The severity index was significantly higher in patients with a high maximum aortic valve gradient (>16 mm Hg) compared to patients with a low maximum aortic valve gradient (≤16 mm Hg), 7.80 ± 1.78 and 3.63 ± 1.35, respectively (Figure 3).

**Table 1:** Characteristics of entire study group.

**Characteristic**	**n = 48**
Age (years, mean ± SD*)	56 ± 9.7
Gender, M/F	32/16
Hypertension, n (%)	32 (67%)
Diabetes Mellitus, n (%)	17 (35%)
Hyperlipidemia, n (%)	20 (42%)
Smoking, n (%)	19 (40%)
Family History of CAD, n (%)	16 (33%)
Transaortic Jet Velocity by Echocardiography, (mean ± SD*)	2.2 ± 1.3
Maximum Aortic Valve Gradient by Catheterization, (mean ± SD*)	26 ± 30
Severity Index, (mean ± SD*)	5.0 ± 2.5
AV Ca (1–6) (mean ± SD*)	3.3 ± 1.7
AV Mobility (1–4) (mean ± SD*)	1.8 ± 0.94

CAD = coronary artery disease; AV Ca = aortic valve calcium; AV Mobility = aortic valve mobility; *SD = Standard Deviation

**Table 2:** Characteristics of patients based on severity of aortic valve disease.

**Characteristic**	**Aortic Sclerosis n = 28**	**Mild-Mod AS n = 12**	**Severe AS n = 8**	**p value**
Age (years, mean ± SD*)	56 ± 8	56 ± 13	59 ± 10	0.37
Gender Male/Female	20/8	8/4	4/4	0.54
Hypertension, n (%)	20 (71%)	8 (67%)	4 (50%)	0.54
Diabetes Mellitus, n (%)	10 (36%)	5 (42%)	2 (25%)	0.74
Hyperlipidemia, n (%)	13 (46%)	4 (33%)	3 (38%)	0.72
Smoking, n (%)	12 (43%)	3 (25%)	4 (50%)	0.45
Family History of CAD, n (%)	11 (39%)	4 (33%)	1 (13%)	0.32
Maximum Aortic Valve Gradient by Catheterization (mean ± SD*) (mm Hg)	7.0 ± 2.8	30 ± 16	83 ± 24	<0.0001
Transaortic Jet Velocity by Echocardiography, (mean ± SD*) (m/s)	1.29 ± 0.27	2.65 ± 0.70	4.54 ± 0.60	<0.0001
Severity Index (0 to 12) (mean ± SD*)	3.38 ± 1.06	6.45 ± 2.12	8.38 ± 1.41	<0.0001
AV Ca (1–6) (mean ± SD*)	2.2 ± 0.78	4.1 ± 1.6	5.5 ± 0.76	<0.0001
AV Mobility (1–4) (mean ± SD*)	1.2 ± 0.43	2.4 ± 0.67	3.3 ± 0.49	<0.0001

CAD = coronary artery disease; AV Ca = aortic valve calcium; AV Mobility = aortic valve mobility; *SD = Standard Deviation

## Discussion

The initial study describing the importance of aortic valve calcium in patients with aortic valve disease was reported by Waller et. al. in 1983 [7]. In this necropsy evaluation of 40 elderly patients, the authors found that the grade of aortic valve calcium correlated with the severity of AS. Rubler et. al. extended these observations by using echocardiography to grade aortic valve calcium in 153 males with aortic valve disease [8]. The presence of moderate to severe aortic valve calcium identified those with severe AS confirmed by cardiac catheterization. The severity index was introduced by Bahler et. al. in 1999 as a new echocardiographic measure of aortic leaflet pathology and includes an assessment of both leaflet mobility and calcification [5]. In this study, the authors found that the severity index was independently associated with more rapid disease progression. However, their study included only patients with AS (aortic valve area ≤ 2.0 cm^2^). In our current study, we sought to validate this new echocardiographic scoring method against both invasive and non-invasive hemodynamic measures of aortic valve disease severity. Also, we included patients with aortic sclerosis, as well as patients with AS. The results show that, across the spectrum of calcific aortic valve disease, the severity index was correlated strongly with both the maximum aortic valve gradient, as measured by cardiac catheterization, and with the maximal transaortic jet velocity, as measured by echocardiography.

An important limitation of this study is that it is cross-sectional. Therefore, while it includes patients with lesser degrees of calcific aortic valve disease, it cannot determine whether the severity index provides useful prognostic information in these patients beyond that provided by more traditional hemodynamic measures. However, one retrospective study has reported that aortic valve thickening (a component of the severity index) is associated with increased risk of progression to AS [9]. Validation of the severity index as a prognostic marker for either future clinical events or progression to AS, could be examined in large, prospective studies that include serial echocardiograms.

## Conclusion

In summary, these results confirm that the severity index correlates with hemodynamic severity of aortic valve disease and may prove to be a useful measure in the evaluation of patients with aortic sclerosis and AS.

## Abbreviations

AS - Aortic Stenosis

SD - Standard Deviation

CAD - Coronary Artery Disease

AV Ca - Aortic Valve Calcium

AV - Mobility Aortic Valve Mobility

## Competing interests

The author(s) declare that they have no competing interests.

## Authors' contributions

Author contributions include the following: (1) conception and design of study: KOB, DMS and MJB; (2) analysis and interpretation of data JT, NB, JR and (3) drafting of the manuscript: KOB, DMS, MJB or revising it critically for important intellectual content (all authors). All authors read and approved the final manuscript.
